# A Geometric Approach towards Inverse Kinematics of Soft Extensible Pneumatic Actuators Intended for Trajectory Tracking

**DOI:** 10.3390/s23156882

**Published:** 2023-08-03

**Authors:** Mahboubeh Keyvanara, Arman Goshtasbi, Irene A. Kuling

**Affiliations:** 1 Reshape Lab, Dynamics and Control Group, Department of Mechanical Engineering, Eindhoven University of Technology, 5600 MB Eindhoven, The Netherlands; i.a.kuling@tue.nl; 2 SDU Soft Robotics, SDU Biorobotics, The Maersk Mc-Kinney Moller Institute, University of Southern Denmark (SDU), 5230 Odense, Denmark

**Keywords:** extensible soft robots, inverse kinematics, hyper-redundancy, trajectory tracking, piecewise constant curvature model

## Abstract

Soft robots are interesting examples of hyper-redundancy in robotics. However, the nonlinear continuous dynamics of these robots and the use of hyper-elastic and visco-elastic materials make modeling these robots more complicated. This study presents a geometric inverse kinematics (IK) model for trajectory tracking of multi-segment extensible soft robots, where each segment of the soft actuator is geometrically approximated with a rigid links model to reduce the complexity. In this model, the links are connected with rotary and prismatic joints, which enable both the extension and rotation of the robot. Using optimization methods, the desired configuration variables of the soft actuator for the desired end-effector positions were obtained. Furthermore, the redundancy of the robot is applied for second task applications, such as tip angle control. The model’s performance was investigated through kinematics and dynamics simulations and numerical benchmarks on multi-segment soft robots. The results showed lower computational costs and higher accuracy compared to most existing models. The method is easy to apply to multi-segment soft robots in both 2D and 3D, and it was experimentally validated on 3D-printed soft robotic manipulators. The results demonstrated the high accuracy in path following using this technique.

## 1. Introduction

Soft robots have shown great potential in many robotics applications that require flexibility and adaptation [1]. Despite the recent progress in soft robotics, motion planning and control remain the main challenges due to the complex nonlinear dynamics of these robots [2]. One possible solution could involve designing an inverse kinematics (IK) model, which could then be integrated with an existing joint-space controller to provide a more-straightforward approach [3].

Solutions to soft robots’ IK have been studied using different approaches. In analytical approaches, an absolute solution for the required degrees of freedom (DoFs) is found using the geometry and kinematics of the robot. However, this solution is not trivial due to these robots’ nonlinear equations and hyper-redundancy. Different models have been proposed to overcome this, the most-famous one being the constant curvature (CC) approximation [2,4,5]. Using this model, the authors in [6] suggested an absolute geometrical solution for a single-segment inextensible continuum arm, showing it to be the most-suitable method for a single-segment robot. Combining a piecewise constant curvature (PCC) model and analytical solutions for multi-segment soft robots can lead to complex mathematics, high computational costs, and many simplifications [7]. For this reason, a less-intricate, yet accurate model is needed to overcome the complexity of the analytical solution.

Numerical solutions are a better alternative for IK solutions where state variables are approximated using iterative or optimization techniques. For example, in [8], the deformations of a soft robot under different actuation loads were simulated using FEM. The main benefit of the FEM is its capability to solve the inverse kinematics of nonuniformly shaped robots and torsional robots [9,10]; yet, the results are dependent very much on the robot itself. This method is also computationally intensive for multi-link systems and real-time control. Only recently in [11], using visual servoing, the authors performed the closed-loop trajectory tracking of a soft robot using FEM. In other numerical methods, such as curvature discretization, the nonlinear behavior of soft robots is approximated by multiple rigid links. In [12], the authors suggested 16 rigid link approximations to model a single-segment polymer actuator. In [13], the more-complicated shape of soft robots was approximated by a rigid link modeling. Here, the strain of the robot during actuation was not taken into account; only bending was modeled, and a solution for extensible soft robots is still missing. Other numerical methods, namely heuristic inverse kinematics algorithms, are famous for low computational costs and the possibility of solving large DoF systems [14]. Reference [15] proposed a new cyclic coordinate descent (CCD) algorithm for soft and redundant robots by solving the shortcomings of the standard CCD algorithms. This method is limited to simulations only and has not been tested with real-time controllers and experimental setups.

Learning methods are also employed to overcome the complexity of the IK problem. Mostly, these techniques are combined with other well-known IK solutions to propose faster solutions [16]. For example, in [17], the authors combined the Jacobian inverse kinematics method with feedforward neural networks to control the multi-link robot and optimize energy usage. In learning approaches, similar to FEM approaches, inverse kinematics can be solved for nonuniform robots. However, a physical experimental setup or a sophisticated dynamic model is required for the data acquisition. In [18], a combination of both model-based (numerical) and learning methods was used to solve the inverse kinematics problem at two levels, but only in 2D.

Leveraging this literature and with the aim of proposing a fast and accurate IK solution for extensible multi-segment 3D soft robots that can be used easily in experimental validations, we propose a geometric inverse kinematics model for 2D and 3D single- and multi-segment extensible soft robots. In the proposed method, each robot segment is modeled with a CC approximation and discretized with multiple rigid links connected together with prismatic and rotary joints. The developed method is mathematically simple, can easily be applied to various types of soft actuators, and has negligible error with respect to the workspace. Increasing the DoFs of the robot by adding segments to the robot is straightforward and does not significantly affect the method’s performance. Using this geometric model, an optimization method was employed to find a configuration for the soft robot, resulting in the end-effector reaching the required position. The optimization method allows for working with the boundary conditions using the redundancy of the robot for different tasks, such as tip angle control and pressure control. Although the pose control of soft actuators has been studied before [19], to the best of the authors’ knowledge, no study has proposed an IK method that can put tip angle control as the second priority. Using this model, one can either control the tip angle of the soft robot with the same priority as the position (same as pose control) or solve the IK so that the tip angle is a second priority and position control is the first priority. Another advantage of the proposed IK model is its application diversity; as the obtained results using this model are geometric variables, it is possible to employ this method with most dynamic models. To express how this IK method can be integrated with dynamic models and can be used for trajectory tracking in experimental setups, a model-based control algorithm is required to assist with controlling the robot on a designed trajectory. Different dynamic models have been presented previously [20,21,22,23,24,25,26]. Since this research proposes the IK method for the PCC model, a model-based control based on this modeling is also needed. Furthermore, an experimental setup with a soft robot control unit was used to show the real-time applications of the models presented in this study.

The overall contributions of this work include the following:

1. A fast and accurate IK model for trajectory tracking of extensible soft robots that is easy to use and efficient for multi-segment soft robots.
2. A new approach towards tip angle control of soft robots on desired trajectories.
3. An overview of implementing the IK model for open-loop and real-time closed-loop control via extensive simulations and experiments.

The organization of this work is as follows. After this Introduction, in Section 2, an overview of the IK method, the dynamic model, and the proposed controller are presented. Then, in Section 3, different numerical simulations on single-segment and multi-segment soft robots are studied and validated. Finally, in Section 4, the results of the experiments are presented, followed by a brief conclusion.

## 2. Materials and Methods

In this section, we first present the formulation of our geometry-based IK modeling framework. Then, the dynamic modeling and also the controllers needed for the model-based control are presented. The material presented in this section is later used in simulations and also experiments. Meanwhile, some notations used in this paper are described briefly.

### 2.1. Notations

Throughout this paper, bold letters are used to present column vectors and matrices, respectively, e.g., $q\in {I\;R}^{n}$ and $M\left(q\right)\in {I\;R}^{n\times n}$. The identity matrix is denoted by $I_{k}\in {I\;R}^{k\times k}$. Furthermore, subscript *d* means the desired trajectory, subscript *i* means the $i^{th}$, subscript *e* means elongation, and subscript *b* means bending. The details of other notations used in this paper are summarized in Table 1.

### 2.2. Inverse Kinematics Model

**Figure 1:**
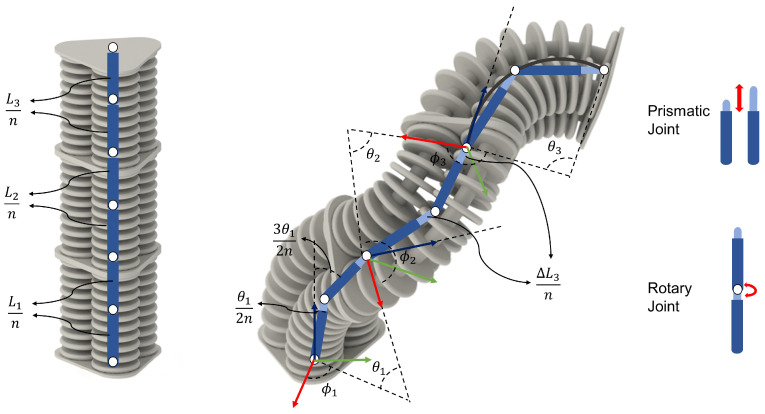
Combination of PCC and rigid link model for the multi-segment soft robot and the respective configuration variables. The configuration before (**left**) and after actuation (**right**) is shown. Each PCC curvature is represented with two rigid links (m=2) presented in dark blue connected to each other with rotary (circles) and prismatic (light blue) joints. Here, n is the number of links in each segment, L is the initial length of the segment, ΔL is the extension of each segment, θ is the bending angle, and ϕ is the deflection angle from the X-axis.

The geometric properties and complex dynamic behavior of soft robots make solving the inverse kinematics more challenging than traditional rigid robots. Our approach to reducing the complexity of soft robots is to discretize the robot curvature with small rigid links, which is also known as the rigid link model. In this model, the rigid links are inextensible and connected via prismatic and rotary joints to cover both extension and rotation in dynamic behavior (Figure 1). The reduced complexity of using this technique enables more-straightforward kinematics solutions for soft robotic applications. Additionally, since many soft robots exhibit uniform shapes with limited torsional motion, the constant curvature approximation is suitable. Therefore, we modeled each segment of the robot as a constant curvature. Then, each curvature was approximated by multiple rigid links for a more-straightforward solution. This approach involves the links in each segment forming a regular polygon due to the constant curvature assumption, enabling a more-accessible geometrical approach to simplify the complex deformation of soft robots.

In the proposed model, the links were connected to each other via two sets of joints: rotary joints to emulate the bending of the actuators and prismatic joints to implement the robot’s extension into the model (Figure 1). Additionally, one primary assumption was that each segment’s rotary and prismatic joints move uniformly. Hence, the overall change in the length of each segment would be the deformation of each prismatic joint multiplied by the number of rigid links, and the same applies to the curvature. Using this method, if we assumed that each segment of a multi-segment soft robot is being approximated by *m* rigid links, and the forward kinematics equation for one segment of this soft robot can be calculated as Equation (1):

(1) $$\begin{array}{c} \left[\begin{array}{c} X_{E_{i}}\\ Y_{E_{i}}\\ Z_{E_{i}} \end{array}\right]=\left[\begin{array}{c} X_{E_{i-1}}\\ Y_{E_{i-1}}\\ Z_{E_{i-1}} \end{array}\right]+\frac{\left(L_{i}+\Delta L_{i}\right)}{m}R_{z}\left(\phi_{i-1}\right)R_{y}\left(\theta_{i-1}\right)\left[\begin{array}{c} cos\left(\phi_{i}\right)\sum_{j=1}^{m}sin\left(\frac{2j-1}{2m}\theta_{i}\right)\\ sin\left(\phi_{i}\right)\sum_{j=1}^{m}sin\left(\frac{2j-1}{2m}\theta_{i}\right)\\ \sum_{j=1}^{ms}cos\left(\frac{2j-1}{2m}\theta_{i}\right) \end{array}\right] \end{array}$$

where $X_{E_{i}}$, $Y_{E_{i}}$, and $Z_{E_{i}}$ are the end-effector positions of the $i^{th}$ segment, $L_{i}$ is the length of the segment, $\Delta L_{i}$ is the change of length of the segment, $\theta_{i}$ is the bending angle of the segment, $\phi_{i}$ is the deflection angle from the X-axis, $R_{y}$ and $R_{z}$ are the rotation matrix around the Y-axis and Z-axis, respectively, and *n* is the number of rigid links in each segment. It is worth mentioning that, although the IK method is presented for a 3D case, Equation (1) is also applicable for 2D systems. The only difference is the deflection angle (ϕ) being zero in the 2D system.

Despite the reduced complexity of the kinematics equation after replacing curvature with small rigid links, it is not possible to solve the problem analytically for the multi-segment robots since the number of variables exceeds the number of kinematic equations. Therefore, after obtaining the forward kinematics, an optimization method is applied to find the parameters of each segment ($L_{i}$,$\phi_{i}$,$\theta_{i}$) in a way that minimizes the error between the desired point, $\left[X_{d}\;\;Y_{d}\;\;Z_{d}\right]$, and the end-effector point derived from the forward kinematics in Equation (1), [$X_{E_{f}}$ $Y_{E_{f}}$ $Z_{E_{f}}$], as in Equation (2). It is worth mentioning that this optimization approach is not limited to only the constant curvature model and can be applied to other soft robotics models.

(2) $$\begin{array}{cc} \min_{\Delta L,\theta ,\phi }\; & {∥\left(\begin{array}{c} X_{d}-X_{E_{f}},Y_{d}-Y_{E_{f}},Z_{d}-Z_{E_{f}} \end{array}\right)∥}_{2}\\ s.t.\; & \Delta L_{i_{min}}<\Delta L_{i}<\Delta L_{i_{max}}\\ \; & \theta_{i_{min}}<\theta_{i}<\theta_{i_{max}}\\ \; & -\pi <\phi_{i}<\pi \end{array}$$

The optimization method applied here was the MATLAB fmincon function, which uses the sequential quadratic programming algorithm (SQP). Since the SQP algorithm cannot find the globally optimized solution, the initial conditions and constraints are essential factors in finding the most-optimal solution for the inverse kinematics. We set the initial conditions for optimization as the configuration variables of the robots at the previous posture to have a smoother and more-energy-efficient solution over the trajectory and avoid abrupt changes in robot posture. In addition, since robots do not have unlimited changes in length or bending, physical constraints were added to the optimization equation to have a more-realistic solution according to the robots’ capability. Equation (2) includes these physical constraints, which are the extension and bending limitations of the robot. As a result of the conditions and constraints, the optimization equations provide a minimum change in the configuration variables for consecutive points on the trajectory. Thus, the energy change and overall energy consumption were minimal.

In the case of a multi-segment robot, as the configuration variables are more than kinematic equations, there are multiple possible solutions for each desired position. These multiple solutions give the robot redundancy, which is beneficial for a secondary task such as tip angle control. Adding additional constraints can include the secondary task in the inverse kinematics solution. This additional constraint for the tip angle control of the 2D robot is presented in Equation (3).

(3) $$\theta_{d}-\sum_{i}^{m}\theta_{i}=0$$

where $\theta_{d}$ is the desired angle and $\theta_{i}$ is the bending angle of each soft segment. With this additional constraint, the IK model benefits from the redundancy to not only find parameters that follow the trajectory, but also calculate the bending angle of each segment in a way that keeps the tip angle at a certain angle. However, the priority of the IK model is to follow the trajectory and, if possible, achieve the second task. Using the boundary condition of the method enabled us to add the secondary task. However, this method limits the IK model to satisfy the secondary task. Therefore, we manipulated the program in a way that the inverse kinematics solution is the priority, and if possible, it follows the second task, which is tip angle control. It is important to mention that it is also possible to add the tip angle control as the first priority and control the position and the angle simultaneously. For other secondary tasks, such as pressure optimization or obstacle avoidance, boundary conditions are still applicable since we needed both inverse kinematics and obstacle avoidance and pressure optimization to happen simultaneously.

### 2.3. Model-Based Control

**Figure 2:**
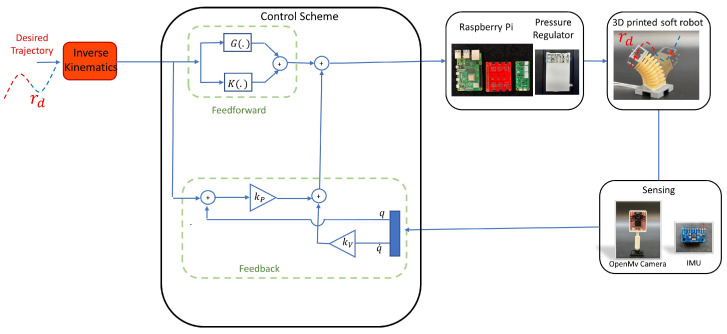
A block diagram representing closed-loop control of the robot on the desired trajectory is presented. Parameters defined in this schematic are later defined in the paper.

Different trajectories were implemented on single-segment and multi-segment soft robots to examine the IK model. To this end, the desired state variables were initially calculated using the IK model. Subsequently, the desired state variables were fed into a model-based controller enabling the robot to be controlled along the intended trajectory. This demonstrated the integration of the IK model with dynamic models and controllers (Figure 2). In this section, the dynamics and also the controller applied to the robot are presented.

For the dynamics of the PCC model, a bishop frame was attached to every point on the backbone curve of the soft robot (in SE(3)), which was parameterized by a state vector $q={\left[\epsilon \;\;k_{x}\;\;k_{y}\right]}^{T}\in R^{3}$. In this context, ϵ represents the strain of the robot, while $k_{x}$ and $k_{y}$ represent the curvature of the robot in the x-z and y-z planes, respectively. The relation between these newly defined state variables and the configuration variables defined in the IK model for each segment (Figure 1) is as follows:

(4) $$\begin{array}{c} \epsilon =\left(L-L_{0}\right)/L_{0}\\ k_{x}=cos\left(\phi \right)\times \theta /L\\ k_{y}=sin\left(\phi \right)\times \theta /L \end{array}$$

Using this notation, the position of each point on the backbone curve is defined as $p\left(q,\sigma \right)=\int_{0}^{\sigma }\Phi \left(q,\eta \right)U\left(q\right)d\eta$, where Φ(q,η) and U describe the differential geometry of the backbone curve. In this context, the equation of motion of the soft robot can be expressed as:

(5) $$\begin{array}{c} M\left(q\right)\ddot{q}+C\left(q,\dot{q}\right)\dot{q}+G\left(q\right)+K\left(q\right)+D\left(\dot{q}\right)=\tau . \end{array}$$

where $M\left(q\right)\in {I\;R}^{n\times n}$ characterizes the system’s inertia, $C\left(q,\dot{q}\right)\dot{q}\in {I\;R}^{n}$ contains the Coriolis and centrifugal forces, and $G\left(q\right)\in {I\;R}^{n}$ denotes the gravitational forces exerted on the robot. Additionally, the hyper-elastic and visco-elastic properties of the robot are described by the two matrices K(q) and $D(\dot{q})$, where the hyper-elastic potential energy can be defined as:

(6) $$\begin{array}{c} U_{elastic}\left(q\right)=\int_{}^{}k_{e}\left(\eta \right)\eta d\eta +\int_{}^{}k_{b}\left(\eta \right)\eta d\eta , \end{array}$$

and here, $k_{e}\left(\eta \right)$ and $k_{b}\left(\eta \right)$ are the elongation and bending stiffness. Using the hyper-elastic potential energy, the stiffness matrix can be calculated as:

(7) $$\begin{array}{c} K\left(q\right)=\frac{dU_{elastic}}{dq} \end{array}$$

Furthermore, a Rayleigh damping matrix is defined as *R*, and so, the damping matrix in the equation of motion is:

(8) $$\begin{array}{c} D\left(\dot{q}\right)=R\dot{q} \end{array}$$

As mentioned previously, there exists a rich literature on the dynamic modeling of PCC models [22,27,28]. In this study, the dynamic model presented in [29] was used for model-based control of the soft robots on desired trajectories. In this modeling, the generalized input vector was chosen as τ(u)=Hu, u being the input pressure to the soft robot and H being a map from the input space to the joint actuation space. *H* was chosen as in Equation (9), where $\gamma_{i}=\left(i-1\right).2\pi /d$ and *d* is the number of pneumatic bellows. Furthermore, $\alpha_{1}>0$ and $\alpha_{2}>0$ are system parameters representing the effective transferal of the differential pressure to the joint forces and will be identified later using experimental data.

(9) $$\begin{array}{c} H=\left[\begin{array}{ccc} \alpha_{1} & \alpha_{1} & \alpha_{1}\\ -\alpha_{2}cos\left(\gamma_{1}\right) & -\alpha_{2}cos\left(\gamma_{2}\right) & -\alpha_{2}cos\left(\gamma_{3}\right)\\ \alpha_{2}cos\left(\gamma_{1}\right) & \alpha_{2}cos\left(\gamma_{2}\right) & \alpha_{2}cos\left(\gamma_{3}\right) \end{array}\right] \end{array}$$

The objective of this research was to achieve trajectory tracking of the soft manipulator in the Cartesian space. Using the inverse kinematics model, the equivalent goal would be for the robot to reach a desired posture in the state space, which theoretically means $\lim_{t\to \infty }q=q_{d}$. It is worth emphasizing that the IK method is independent of the controller. Any controller that works in the state space can be used with this IK method, which gives this method priority over learning methods. As mentioned earlier in Equation (5), the two matrices of K(q) and $D(\dot{q})$ characterize the hyper-elastic and visco-elastic properties of the system. Due to this property of soft robots, as discussed in [30], soft robots have a self-stabilizing feature that proves to be advantageous for controlling purposes. With this analysis, the controller used for trajectory tracking is:

(10) $$\begin{array}{cc} \tau & =\ddot{q}+K\left(q_{d}\right)+G\left(q_{d}\right)\\ where,\ddot{q} & =\ddot{q_{d}}-k_{v}\left(\dot{q_{d}}-\dot{q}\right)-k_{p}\left(q_{d}-q\right) \end{array}$$

where the two terms of $K(q_{d})$, the stiffness matrix, and $G(q_{d})$, the gravity matrix, are the feedforward terms and $\ddot{q}$ was chosen so that the feedback controller was a PD controller. Here, $q_{d}$ is the desired trajectory in the state space, which was designed using the inverse kinematics model. Furthermore, $\ddot{q_{d}}=0$, and as the trajectory tracking was assumed to be slow, $\dot{q_{d}}=0$. The graphical interpretation of this controller is pictured in Figure 2.

## 3. Simulations

Using the proposed methods in Section 2, a set of simulations that can demonstrate how the IK method can be used for trajectory tracking of soft robots is presented. The Github code for the IK simulations is available at: https://github.com/MahboubehKeyvanara/Inverse-Kinematics-of-Soft-Robots (accessed on 11 May 2023).

### 3.1. Single-Segment Soft Robot

**Figure 3:**
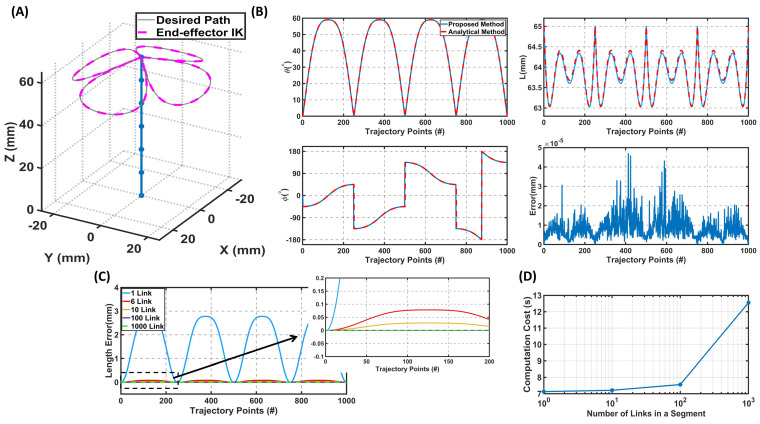
The inverse kinematics model results for a single-segment soft robot over a given trajectory divided into 1000 points. The curvature of the robot is divided into 6 small rigid links. (**A**) The soft robot’s configuration (robot is shown in blue) and calculated end-effector position using the IK model. (**B**) The calculated parameters at different points of the trajectory (θ, ϕ, *L*) using the IK model and analytical solution and the error between the desired position and the IK model over the trajectory. (**C**) The error between the calculated length in analytical solutions from the proposed method for various numbers of links. (**D**) The computational cost of solving the inverse kinematics model for the 1000 points versus the number of rigid links in each segment. By decreasing the number of links, the computational cost decreases, but the length error increases considerably.

In the first step, the proposed inverse kinematics solution was applied to a single-segment 3D soft robot that follows a four-sided flower trajectory, as shown in Figure 3. Since an analytical solution is possible in this case, the obtained results from our method can be compared with analytical parameters for validation. Here, the analytical solution is from [31]. As presented in Figure 3, the IK model followed the desired trajectory with an average error of $8.41\times 10^{-6}$ mm, which is less than 0.001% of the workspace. It can also be seen that the calculated bending and deflection angles were identical for both the analytical and presented IK models. The only difference between these two solutions was the length of the robot. This difference was expected as the actual curvature is longer than the rigid link approximation. A possible solution to overcome this difference is, as shown in Figure 3C, to increase the number of links, which considerably decreased this error. For instance, modeling each segment with 10 links resulted in an error of 0.035 mm, which is less than 0.05% of the robot’s length. By increasing the number of links from 10 to 100, the error was reduced to almost zero. Therefore, increasing the number of links, as expected, reduced the error in length.

However, increasing the number of links increased the computational times. For example, the computational cost for ten links and 1000 points over the given trajectory was 7.21 s (Figure 3D), while with one link, it was 7.13 s and 7.56 s for 100 links. Although the computational cost increased with more links, this increase was negligible compared to the effect on the length error. In addition, the average calculation time for each point was 7.21 ms for the ten-link scenario, which is less than reported for kinematics in similar studies such as [32].

### 3.2. Multi-Segment Soft Robot

**Figure 4:**
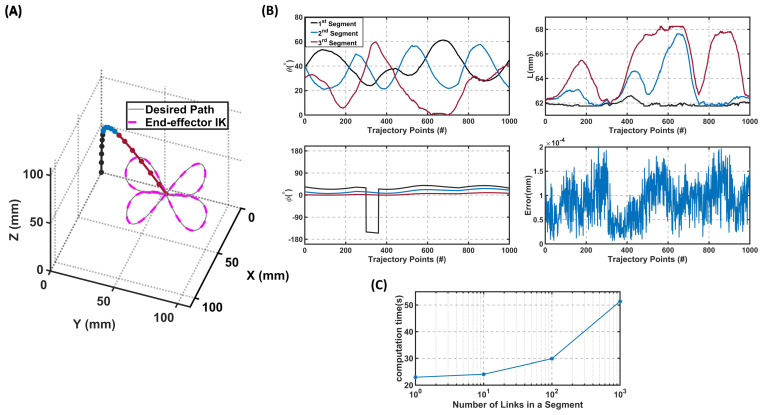
The inverse kinematics model results for a three-segment robot, where each segment is modeled with six small rigid links. (**A**) The robot configuration and calculated end-effector position using the IK model. The first segment of the robot is shown in blue, the second segment in black, and the third segment in red. (**B**) The calculated parameters (θ, ϕ, *L*) of all segments using the IK model and the error between the desired position and the IK model solution. The average error in this case is $1.95\times 10^{-5}\%$ of the workspace. (**C**) The computational time of the inverse kinematics versus the number of links in each segment. Video available at: https://youtu.be/Tl1P8RlE88A (accessed on 11 May 2023).

Unlike for the single-segment robot, the analytical solution is not possible for multi-segment soft robots since the number of DoFs exceeds the number of kinematics equations; thus, the calculated parameters cannot be compared with a particular result. Here, we studied a three-segment soft robot tracking a 3D trajectory, as shown in Figure 4. The proposed IK method generated a continuous path for the three-segment soft robot even though the path was quite complex in 3D. As can be seen, the average error between the end-effector and the desired position over the given trajectory was $1.05\times 10^{-5}$ mm. This small error is lower than the reported error in similar prior work [7,17]. In addition, as shown in the calculated parameters (Figure 4B), there were no abrupt changes in the change of length, the bending angles, or the deflection angle, which led to the smooth motion of the robot over the given trajectory.

**Figure 5:**
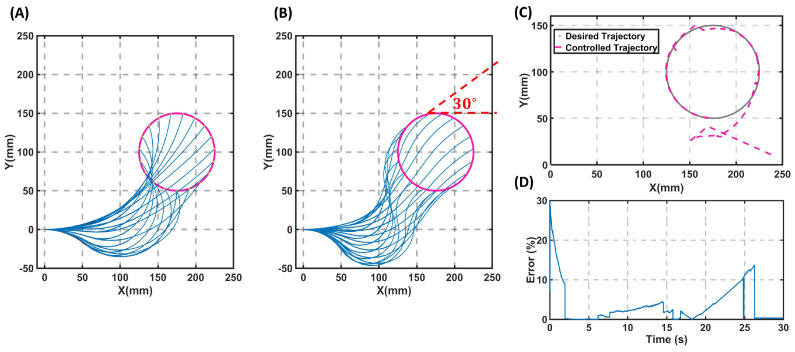
Comparison of two different IK solutions on the same trajectory for a four-segment planar soft robot. (**A**) The robot only has the task of following the desired trajectory of a circle (the robot is shown in blue and the trajectory is shown in pink). (**B**) The robot has a second task of keeping the tip angle a constant value of $30^{\circ }$ with respect to the horizon while tracking the circle. (**C**) The simulation results of the robot being controlled on the desired trajectory pictured in (**B**); the grey line is the desired trajectory, and the pink dashed line is the trajectory followed by the robot. The initial state of the robot is $q_{0}=\left[0,0,0,0,0,0,0,0\right]$. (**D**) The norm of error between the desired trajectory and the position of the end-effector.

Moreover, using the additional constraint defined in Equation (3), it is also possible to design trajectories for a robot with a constant tip angle. To further study this, a four-segment 2D soft robot followed the desired trajectory of $X_{d}=175+50cos\left(t\right)$ and $Y_{d}=100+50sin\left(t\right)$. This trajectory was given as the input to the IK code. The IK method was employed without the additional tip angle constraint in Figure 5A. It can be seen that the state variables were calculated at every step so that the change of these variables with respect to the previous step on the trajectory was minimum, and hence, the energy consumption was minimum. This is a key factor in generating smooth trajectories without unwanted jumps in the robot’s state variables. Since, in this example, the robot had redundancy with respect to the task space, except for following the desired trajectory, it was possible to add tip angle constraints on the robot with lower priority. As explained in Section 2.2, an extra constraint was added to the robot’s trajectory with the aim of having a constant tip angle throughout the trajectory tracking. Figure 5B depicts how different the robot moved as it was constrained to keep the tip angle constant.

It has been shown that the IK model is able to generate smooth 2D and 3D results even on complex trajectories. The next step was to demonstrate how it can be used as an input to dynamic models. To be able to control the robot on the desired trajectories, the physical and material parameters of the robot need to be identified. The soft manipulator used in this research had the following physical parameters: the mass $m_{0}=17.3$ g, and the relaxation length of $l_{0}=64.4$ mm. Considering preliminary uni-axial tension tests, the 3D-printed elastomer material was estimated to be linear isotropic with a Young’s modulus of E=80 MPa and a Poisson’s ratio of ν=0.49. Considering the geometry and material of the soft robot, the hyper-elastic and visco-elastic material parameters and the Rayleigh damping matrix were chosen similarly to [29] (their Table 1), and no further identification was required.

For the first simulation, a circular trajectory was chosen for the four-segment 2D soft robot (Figure 5). Here, using the IK model, the desired state variables for each robot segment were calculated, and using the model-based controller, the robot was controlled on the desired trajectory. To control the robot, a combination of feedforward and feedback control, as explained in Section 2.3, was employed. For the sake of brevity, only the results of the controller applied to the trajectory of Figure 5B are reported in Figure 5C,D. The PD controller used in this case was formulated as $\tau =M\times \left[\ddot{q}-k_{v}\times \left(\dot{q_{d}}-\dot{q}\right)-k_{p}\times \left(q_{d}-q\right)\right]+C\left(q,\dot{q}\right)+D\left(\dot{q}\right)+K\left(q_{d}\right)$ with $k_{p}=80\times I_{8}$ with $k_{v}=3\times I_{8}$. The robot started tracking the trajectory from the initial state of zero, and as shown in Figure 5D, the norm of the error of following this trajectory was less than 12%. It is important to emphasize that the selected trajectory for the robot posed a significant challenge due to the consistent tip angle of the robot. As a result, controlling a four-segment soft robot on this particular trajectory is demanding. Yet, the results presented in Figure 5 verified that the IK model generated smooth trajectories and controlling a multi-segment soft robot on a trajectory with a second desired task did not have abrupt changes. Compared to existing learning methods such as [17], the presented approach also had the advantage of being independent of the controller used.

## 4. Experiments

So far, various simulations have been utilized to demonstrate the results of the IK model. This section, however, is dedicated to showcasing the practical application of the model through a series of experiments.

### 4.1. Experimental Setup

An experimental setup, including a soft robot and a hardware system to control the robot, was developed to show how the IK model and designed controllers can be implemented on a soft robot. Different experiments were run using single-segment and multi-segment soft robots. The single-segment robot is presented in Figure 2. Each soft segment comprised three parallel embedded pneumatic bellows. By inflating or deflating each bellow, the segment can change its posture. Actuating all the bellows simultaneously results in a change in the robot’s length. Moreover, by pressurizing the bellows unequally, the robot can form a pose that produces a curve with constant curvature. Hence, this robotic system was suitable for experiments in this research case, as it can be a PCC model.

A soft robotic control unit (SRC), as in [33], was used for fast and accurate model-based control. In this control unit, the control and data acquisition were performed using a Raspberry Pi 4 (2GB RAM). A proportional piezo-actuated pressure regulator (Festo, VEAB-L-26-D7-Q4-V1-1R1) with a custom Raspberry Pi Hat simultaneously allowed pressure measurements. The main software of this setup was written in Python and designed to read multiple sensors, simultaneously control several VEAB regulators, and communicate with other devices via TCP/IP. Due to large continuous deformations and potential changes in the dynamics, sensing and adding sensors is quite challenging in soft robotics. For this reason, to record the angular deflections of the soft robot, a 9-DoF inertial measurement unit (Bosch, BNO055) was attached to the robot’s end-effector. The IMU sensor contained a 3-DoF gyroscope, a 3-DoF accelerometer, and a 3-DoF magnetometer. A Kalman filter was applied to the sensor data and filtered the noise and other inaccuracies from the measurements. Raw IMU data (including the measurements of acceleration, magnetic orientation, and angular velocity) were processed onboard by the embedded microprocessor in the BNO-055 sensor and output as roll–pitch–yaw (RPY) angles. The information from the IMU sensors was read directly from the SRC software and could be used throughout the real-time control of the soft robot. This control setup connected the sensor via $I^{2}C$ to the SRC. In order to read the Cartesian movements of different points on the backbone curve of the soft robot, an OpenMV H7 R2 camera was employed. Using colored markers and color-detection methods, the position of the markers on the backbone was recorded, and with that, the length of the robot was determined. Hence, all the state variables of the robot can be known throughout the experiments using both vision (strain) and the IMU sensor (bending). In this setup, the regulators had a 100–150 ms delay, and the control rate could reach a max of 300–400 Hz. The overview of the design of the real-time control of soft robots is depicted in Figure 2.

First, the parameters for the mapping matrix defined in Equation (9) were identified to start the experiments. A series of experiments was run to identify the mapping matrix. In each experiment, a set of quasi-static pressures was added to the robot. With each set of pressure, the robot reached a final point in the Cartesian space. This point could be identified using the data from the OpenMV camera (${\left[X,Y,Z\right]}_{markers}$) and IMU sensors ([roll,pitch,yaw]). Through this calibration, the state variables of the dynamic model were identified. These state variables were fed into the model, and the required input pressures for the robot were calculated via the simulations, which were then compared with the pressure input of the experiments. With this comparison, the parameters were identified for the mapping matrix of Equation (9), as $\alpha_{1}=8\times 10^{-4}$ and $\alpha_{2}=7.91\times 10^{-7}$. It is worth mentioning that, since the IMU sensors were also used for each single-segment soft robot, only one position dataset from the cameras was sufficient to find the state variable. Here, we used $Z_{markers}$.

### 4.2. Results

Here, the results of four experiments performed using single-segment and multi-segment soft robots are presented. Specifically, two experiments were conducted using a single-segment soft robot by employing the control block diagram shown in Figure 2, and two experiments were performed using a two-segment soft robot.

**Figure 6:**
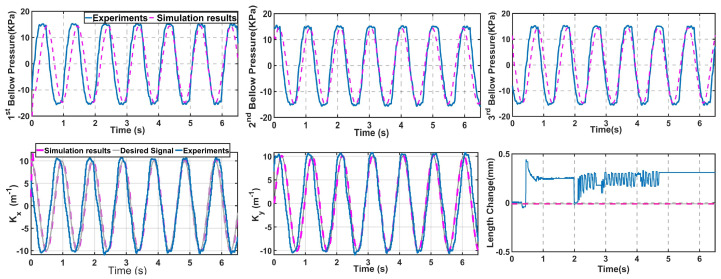
Experimental tracking results for a single-segment soft robot on a circular trajectory. (**Top** row) Pressure values; dashed lines are the designed values, and blue lines are the regulator measurements. (**Bottom** row) State variables of the robot in two different planes indicating the state variables of the dynamic model. The grey plots are the results of the IK model, which are the desired trajectory; the dashed lines show the simulation results of the controller, and the blue lines show the robot’s movements in the experiments.

For the first experiment, a controller was designed to keep the single-segment robot’s end-effector on the trajectory of $X_{d}=20cos\left(6t\right)$, $Y_{d}=20sin\left(6t\right)$, $Z_{d}=60$. In every step of the real-time control, first, the control of the robot was simulated using the feedback controller presented in Equation (10). At this step, there was a set of three pressures that indicated how much each bellow needed to be pressurized to follow the desired trajectory. These values were fed directly into the robot using pressure regulators. As the robot was pressurized, the IMU sensors and camera data sent feedback to the model to help calculate the error with respect to the desired state variables. From there, a new set of pressure signals was calculated. The designed controller in this study was programmed in MATLAB/Simulink and communicated via TCP/IP with the SCR unit at 250 Hz. As the experiments were run, it could be verified that the robot could follow the desired trajectory. Figure 6 (top row) pictures the designed values for the pressure of each bellow and compares them with the pressure produced by the regulators in each bellow. It depicts how these two values overlapped after the initial transient state was finished, meaning the experimental setup generated the pressure inputs very close to the designed pressure values, which helped the trajectory tracking for the soft robot. Furthermore, using the data from the camera and the IMU sensors, it was possible to study the state variables of the robot as it tracked the trajectory. Figure 6 (bottom row) compares these state variables between the designed values from the IK solution, the model simulations, and the controlled values in the experiments. This figure also verifies that trajectory tracking could be achieved with combining the IK model, the dynamic model, and the experimental setup.

**Figure 7:**
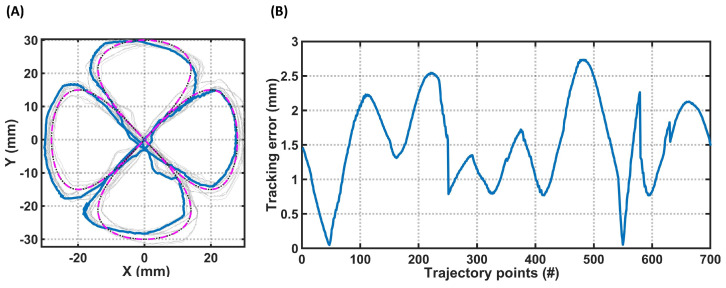
Experimental tracking results for a single-segment soft robot on a four-sided flower. (**A**) Top view of the control of the robot on the designed trajectory of Figure 3. The dashed pink plot is the desired trajectory, and the grey plots are different experiments. The continuous blue plot is the average of the trajectory followed by the robot. (**B**) The norm of the error between the trajectory followed by the tip point and the desired one.

For the second experiment, a controller was employed to control the robot on the desired trajectory previously presented in Figure 3. The trajectory was added to the dynamic model, and the desired pressures were added to the robot to perform the desired trajectory tracking. The top view of the final trajectory tracking of the robot is presented in Figure 7A. The error between the desired trajectory and the following one was less than 3 mm (Figure 7B), which is acceptable considering the trajectory’s complexity for a three-bellowed robot. In this experiment, as shown in Figure 3, the robot experienced a length change, which verified the IK model’s validity for trajectories that involve a change in the length of the robot. For both experiments, the initial condition was zero, $q_{0}=\left[0,0,0\right]$, and the control parameters were chosen as ${\mathrm{kv}}_{p}=0.16\times I_{3}$ and $k_{v}\approx 0\times I_{3}$.

**Figure 8:**
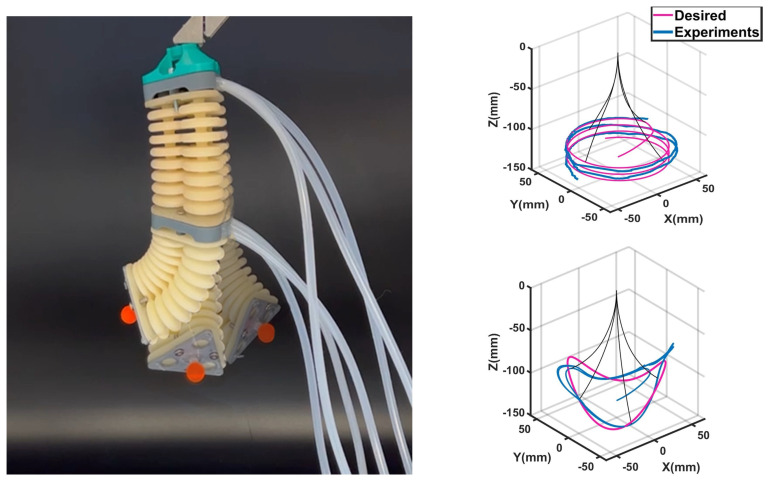
Experimental tracking results for a multi-segment soft robot. On the **left**, a 3D-printed two-segment soft robot is used for the experiments. Each segment of the soft robot has three bellows. The red marker is used for trajectory tracking of the robot’s end-effector. The robot is mounted in the −Z direction to compensate for gravity. On the **right** are experimental results for the desired spiral trajectory and saddle trajectory. The black lines show the robot’s shape in the simulations (four positions are superimposed in the image). The pink trajectories show the desired trajectory, and the blue trajectories show the followed trajectory by the robot (Supplementary Material).

Multiple experiments were also conducted to demonstrate that the IK model is compatible with dynamic models for multi-segment soft robots. For these experiments, a two-segment soft robot was employed. This robot is shown in Figure 8. The robotic setup was composed of two single-segment robots identical to the previous experiments. The connecting parts to these robots were fabricated using a stereolithography printer (ELEGOO Saturn2). The air tubes were connected to each segment separately using these connecting parts, and the pressure inside each bellow was controlled using six pressure regulators. Two experiments were conducted using this setup. These experimental validations were particularly important as there is limited work in the current literature on the IK of multi-segment extensible soft robots in 3D. Here, in the first experiment (Figure 8), the robot was controlled to follow a spiral trajectory with a Cartesian equation of $X_{d}=0.05cos\left(0.4t\right)$, $Y_{d}=0.05sin\left(0.4t\right)$, $Z_{d}=-0.11-0.0005t$, and in the second experiment, it was controlled to follow a hyperbolic paraboloid trajectory with the equation of $X_{d}=0.05cos\left(0.4t\right)$, $Y_{d}=0.05sin\left(0.4t\right)$, $Z_{d}=-0.11-0.05sin\left(0.4t\right)cos\left(0.4t\right)$. In both experiments, the simulations were run to design the required controllers and were then added to the robot to perform open-loop experiments. The initial condition of the robot was the zero state. These trajectories were chosen due to their complexity for IK calculations and control. As can be seen in these figures, the IK model generated a continuous smooth trajectory in 3D for the two-segment soft robot. It is important to mention that generating more-complex controllers can improve the robot’s trajectory tracking, and the errors in these figures were not due to the deficiency of the IK model, but the employment of open-loop control.

## 5. Conclusions

The objective of this study was to develop an inverse kinematic model for soft extensible actuators using a piecewise constant curvature approach, applicable to both single-segment and multi-segment configurations in both 2D and 3D environments. In this method, each segment of the soft actuator was modeled with multi-rigid links, which were connected through rotatory and prismatic joints. The approach used a simple procedure to minimize the error between the desired position of the end-effector and the forward kinematics equations. In this energy-efficient method, adding a second desired task, such as tip angle control, is possible using additional constraints. The presented method was verified through simulations and different experimental results, which validated the method’s applicability to different trajectories. One main advantage of the presented method is the possibility to use any controller for the robot, independent of the IK method itself. This sets it apart from learning methods, as it gives the flexibility to choose a controller that best suits the problem at hand. Regardless of the type of controller and the dynamic model, the IK model has a low computational cost and high accuracy for trajectory tracking. For future work, we will focus on achieving a more-comprehensive range of models for the soft robot and propose a model that is not only limited to PCC. Furthermore, we will use other optimization methods, such as a generic algorithm, to find the globally optimized points, which could result in higher precision. To conclude, this research provided a fast and accurate inverse kinematics model for trajectory tracking of extensible soft robots, a new approach toward tip angle control, and an overview of its implementation for real-time control.

## Figures and Tables

**Table 1:** Summary of the mathematical notation used in this paper.

Symbol	Description
$X_{E_{i}},Y_{E_{i}},Z_{E_{i}}$	Position of end-effector of the $i^{th}$ soft segment
$L_{i}$, $\Delta L_{i}$, and $\theta_{i}$	Length, change of length, and bending angle of the $i^{th}$ soft segment
$\phi_{i}$	Deflection angle of the $i^{th}$ soft segment with respect to the X-axis
$R_{y}$ and $R_{z}$	Rotation matrix with respect to the Y-axis and Z-axis, respectively
*m*	Number of soft segments in a multi-segment soft robot
q	State variable used in dynamic model
$q_{d}$	Desired state variables (trajectory) designed using IK model
ϵ, $k_{x}$, and $k_{y}$	Strain of the robot and curvature of the robot in the x-z and y-z planes
*n*	State dimensions for the soft robot
$M\left(q\right)\in {I\;R}^{n\times n}$	System’s inertia
$C\left(q,\dot{q}\right)\dot{q}\in {I\;R}^{n}$	Matrix of Coriolis and centrifugal forces
$G\left(q\right)\in {I\;R}^{n}$	Matrix of gravitational forces exerted on the robot
K(q) and $D(\dot{q})$	Matrices of hyper-elastic and visco-elastic properties of the robot
$U_{elastic}\left(q\right)$	Hyper-elastic potential energy
u	Input pressure to the soft robot
H	Map from the input space to the actuation space of the robot
$\alpha_{1}$, $\alpha_{2}$	Effective transferal of differential pressure to joint forces
$k_{p}$ and $k_{v}$	Controller gains designed for trajectory tracking

## Supplementary Materials

The following supporting information can be downloaded at: https://www.mdpi.com/article/10.3390/s23156882/s1, Video S1: Inverse kinematics of soft extensible actuators.
